# Insights into antiradical mechanism and pro-oxidant enzyme inhibitor activity of walterolactone A/B 6-*O*-gallate-β-d-pyranoglucoside originating from *Euonymus laxiflorus* Champ. using *in silico* study†

**DOI:** 10.1039/d2ra05312h

**Published:** 2022-10-19

**Authors:** Phan Tu Quy, Nguyen Anh Dzung, Mai Van Bay, Nguyen Van Bon, Doan Manh Dung, Pham Cam Nam, Nguyen Minh Thong

**Affiliations:** Department of Natural Sciences & Technology, Tay Nguyen University Buon Ma Thuot 630000 Vietnam; Institute of Biotechnology and Environment, Tay Nguyen University Buon Ma Thuot 630000 Vietnam; The University of Danang – University of Science and Education Danang 550000 Vietnam; The University of Danang – University of Science and Technology Danang 550000 Vietnam; The University of Danang – Campus in Kon Tum 704 Phan Dinh Phung Kon Tum Vietnam nmthong@kontum.udn.vn

## Abstract

The ability of a new compound, Wal, (walterolactone A/B 6-*O*-gallate-β-d-pyranoglucoside) originating from *Euonymus laxiflorus* Champ. as a hydroperoxyl radical scavenger and pro-oxidant enzyme inhibitor was studied *in silico*. Different mechanisms, reaction locations, and chemical species of Wal in aqueous solution were taken into consideration. Formal hydrogen transfer from the OH group has been discovered as the chemical process that contributes most to the antioxidant properties of Wal in nonpolar and aqueous solutions. The overall rate coefficients for polar and non-polar environments are expected to have values of 7.85 × 10^6^ M^−1^ s^−1^ and 4.84 × 10^5^ M^−1^ s^−1^, respectively. According to the results of the investigation, Wal has greater scavenging activity against the HOO˙ radical than the reference antioxidant Trolox at physiological pH (7.4). In addition, docking results indicate that Wal's antioxidant properties involve the inhibition of the activity of enzyme families (CP450, MP, NO, and XO) that are responsible for ROS production.

## Abstract 2

*In silico* evaluation of a novel compound isolated from *Euonymus laxiflorus* Champ. for its ability as a hydroperoxyl radical scavenger and pro-oxidant enzyme inhibitor.

## Graphical abstract

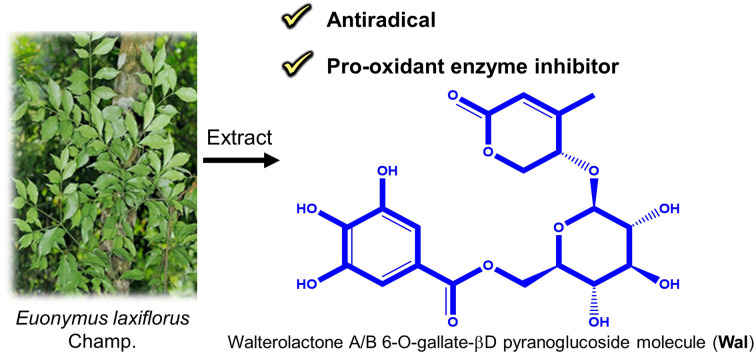

## 1. Introduction

The presence of elevated quantities of free radicals in the human body can cause disruptions in the body's metabolic processes, which can then lead to a variety of disease-causing processes.^1,2^ During the process of intracellular metabolism, the formation of reactive oxygen species (ROS) is catalyzed by five different enzymes, such as: cytochrome P450 (CP450), lipoxygenase (LO), myeloperoxidase (MP), NADPH oxidase (NO), and xanthine oxidase (XO).^3^ To reduce oxidative stress and sustain biochemical redox equilibrium, antioxidants in the cell must work as well as they should. Antioxidant molecules can be created in the body or taken with food. They play an important function in the regulation of ROS formation, which helps keep redox homeostasis and also provides defense from oxidative destruction. Finding new antioxidants is a topic that receives a significant amount of attention in the scientific community.

**Fig. 1:**
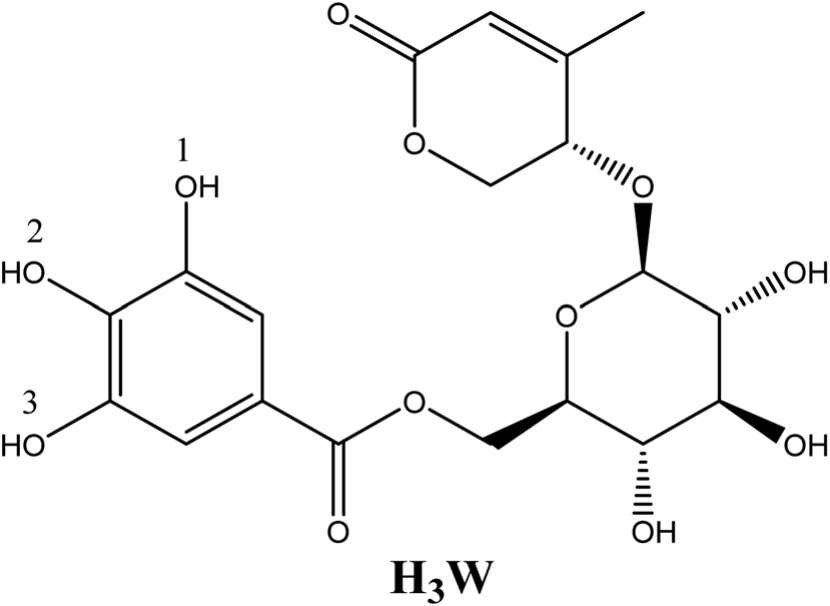
Structure of walterolactone A/B 6-*O*-gallate-β-d pyranoglucoside molecule (Wal).

The medicinal plant *Euonymus laxiflorus* Champ. is a member of the Celastraceae family. In Vietnam's Central Highlands, ethnic minorities have employed it in traditional Vietnamese medicine. Recent research demonstrates that the chemical compounds extracted from *Euonymus laxiflorus* Champ. have powerful biological benefits, including antidiabetic and antioxidant capabilities.^4,5^ Notably, the walterolactone A/B 6-*O*-gallate-d pyranoglucoside molecule (Wal) (Fig. 1), which was derived from this plant, was a novel ingredient with numerous biological activities.^4^ The experimental examination suggests that this chemical is an effective antioxidant.^4^ However, no data on the effect of solvent polarity, the role of its various acid–base species, or reaction mechanisms on this compound's radical scavenging activity have been reported. More research on the compound Wal is required to fully comprehend its potent antioxidant properties.

Recent advances in computing power, software, and computational chemistry methodologies have resulted in the provision of highly accurate information as well as a huge reduction in time and cost compared to experimental methods.^6^ Consequently, *in silico* investigations are regarded as an efficient method for predicting the structural features, biological activity, and reaction processes of new compounds.^7,8^

This research was carried out with the intention of accomplishing the following three primary objectives: (1) determining which of two possible mechanisms for Wal's antioxidant activity involving FHT and SET is the most likely; (2) calculating an approximation of radical scavengers behavior by carrying out a kinetic analysis of the reaction that takes place between the HOO˙ radical and Wal under physiological conditions; and (3) figuring out if there is a direct connection between compound Wal and enzymes in the body that make ROS.

## 2. Computational methods

### 2.1. Quantum chemical calculations

For geometry optimizations and frequency computations, the density functional theory (DFT) techniques provided by the software Gaussian 09 were used.^9^ According to prior publications, the DFT method at the M06-2X/6-311+G(d,p) level of theory was utilized effectively for thermodynamic and kinetic calculations involving radical processes.^10,11^ SMD (solvation model density) was utilized for estimating the influence of pentylethanoate and water medium on the antiradical capability of an antioxidant. Based on the QM-ORSA (quantum mechanics based test for overall free radical scavenging activity),^12^ kinetic calculation techniques were implemented in accordance with the transition state theory (TST) and the standard model (1 M, 298.15 K).^13,14^ Table S1 in ESI† provides more information about the method. For the species that have multiple conformers, all of these were investigated and the conformer with the lowest electronic energy was included in the analysis.^15^ The hindered internal rotation treatment was also applied to the single bonds to ensure that the obtained conformer has the lowest electronic energy.^16,17^

### 2.2. Molecular docking simulations

Utilizing AutoDock Tools^18^ and AutoDock Vina,^19^ simulations of molecular docking were conducted. The structures of ROS-generating enzymes such as CP450, LO, MP, NO, and XO (corresponding to the codes 1OG5, 1N8Q, 1DNU, 2CDU, and 3NRZ) are available in the Protein Data Bank. The commercially available drugs, such as 5-fluorouracil (FLU),^20^ zileuton (ZIL),^21^ melatonin (MEL),^22^ dextromethorphan (DEX),^23^ and febuxostat (FEB)^24^ were utilized as controls for evaluation of each receptor inhibitor activity, respectively. The validation process identified the critical protein–ligand interactions that may positively contribute to antioxidant activity, in accordance with previous studies.^25–28^ Using the application Discovery Studio, the interactions between ligand and receptor are examined.^29^

## 3. Results and discussions

### 3.1. DFT analysis of HOO radical scavenging in physiological contexts

#### 3.1.1 Dissociation states of Wal

Our prior research demonstrated that the chemical species of antioxidants have a substantial effect on their antiradical action under physiological settings.^30–32^ In order to identify the dissociation states of Wal in aqueous environments, the p*K*_a_ value was calculated by isodesmic processes and thermochemical cycles.^33,34^

**Fig. 2:**
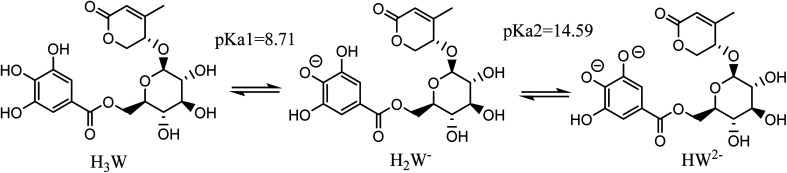
The possible deprotonation paths of Wal at physiological pH.

Taking into account all of the potential routes of deprotonation (see Fig. 2), Wal is polyphenol with three OH functional groups, and its dissociation in water follows this order: At p*K*a_1_ = 8.71, the first proton loss process occurred at the OH in position 2, producing an H_2_W^−^ anion; the second deprotonation step at p*K*a_2_ = 14.59, associated with the OH in position 1, created the HW^2−^ specie. Finally, the W^3−^ specie was formed by the H^+^ loss of the benzene ring's final OH group, and the relative p*K*a_3_ value was 25.11. In water solvent at pH = 7.4, only neutral (H_3_W) and anion (H_2_W^−^) forms of Wal exist in molar fractions (*f*) of 0.942 and 0.058, respectively (see Table 1).

**Table 1:** Molar fractions of various acid–base states of Wal at pH = 7.4

Species	H_3_W	H_2_W^−^	HW^2−^
*f*	0.942	0.058	0

#### 3.1.2 Thermochemical analyses

Several commonly used mechanisms for evaluating the radical scavenging ability of antioxidant compounds are FHT – formal hydrogen transfer, SETPT – single electron transfer followed by proton transfer, SPLET – sequential proton loss electron transfer, and RAF – radical adduct formation.^35^ Regarding SETPT and SPLET, these chemical routes exist only in the polar environment, corresponding to a single electron transfer process from various acid–base species, including neutral and anionic species (the first step of SETPT, the second step of SPLET). As a result, both SETPT and SPLET can be reduced to single electron transfer (SET) routes. Furthermore, previous studies have shown that a localized C=C bond and an aromatic ring are unfavorable to the RAF process.^36–38^ Therefore, in this work, the FHT and the SET were chosen as the two primary mechanisms to clarify the link between the structural features of Wal and its antioxidant activity.

As noted previously, all the states formed from the acid–base equilibria in a water solvent need to be considered for evaluation of the HOO˙ radical scavenging capacity of Wal*via* two key mechanisms as follows (eqn (1)–(4)):

FHT (Formal Hydro Transfer)

1 H_3_W + HOO˙ → H_2_W˙ + H_2_O_2_

2 H_2_W^−^ + HOO˙ → HW˙^−^ + H_2_O_2_

SET (Single Electron Transfer)

3 H_3_W + HOO˙ → H_3_W˙+ + HOO^−^

4 H_2_W^−^ + HOO˙ → H_2_W˙ + HOO^−^

To test the viability of any chemical process, the Gibbs free energies (Δ*G*^o^) for the reaction between the HOO˙ radical and all states were initially estimated *via* the FHT and SET routes. Because ionic species are unstable in the pseudo-lipid environment (corresponding to pentyl ethanoate solvent), only neutral ones acting in accordance with the FHT route (excluding SET) were investigated. Table 2 shows the computed Δ*G*^o^ values for all reaction channels in nonpolar and polar solutions, respectively.

**Table 2:** The Gibbs free energies (Δ*G*^o^) of the modeled channels in lipid and aqueous media (in kcal mol^−1^)

Pathways	Aqueous medium		Lipid medium
	H_3_W	H_2_W^−^	H_3_W
FHT, site 1	−0.82	−13.46	0.63
FHT, site 2	−6.14	—	−5.43
FHT, site 3	−1.47	−12.56	−0.06
SET	33.61	5.25	—

In a pentylethanoate solvent containing only the H_3_W form, which is explored here for the FHT mechanism, the Δ*G*^o^ values when the HOO˙ captures hydrogen atoms at sites 2 and 3 are exergonic by −5.43 and −0.06 kcal mol^−1^, respectively, but it is only slightly positive at site 1 (Δ*G*^o^ = 0.63 kcal mol^−1^). In contrast, in aqueous solution, all investigated reactions for H_3_W and H_2_W^−^ are predicted to be endergonic. The range of Δ*G*^o^ values from −0.82 to −13.46 kcal mol^−1^ proves that the FHT route is thermodynamically favorable.

Regarding the SET route in aqueous solution, the computed Δ*G*^o^ values of all available states were reported to be significantly endergonic. As can be seen in Table 2, these values were 33.61 and 5.25 kcal mol^−1^, corresponding to H_3_W and H_2_W^−^ forms, respectively. This finding suggests that SET is an unspontaneous process. Consequently, the SET route has no effect on Wal's overall reactivity to the hydroperoxyl radical.

#### 3.1.3 Kinetic analyses

Although spontaneity is a crucial factor in determining chemical reactivity, it is known to be not always enough. This is because an exergonic reaction can occur at either fast or slow speeds. Consequently, studying the kinetics involved in the antioxidant action of chemical substances is essential for accurately anticipating this behavior. Not only must an effective antioxidant be capable of spontaneously reacting with free radicals, but it also needs to be quicker than the protein targets that it is designed to defend. As a result, the rate constants of the exergonic reaction channels have indeed been calculated in this article. Those with Δ*G*^o^ greater than zero were omitted from the kinetic investigation, since they would be so easily reversible, even if they occurred at large rates, the effects on the antioxidant capacity would be insignificant.

**Fig. 3:**
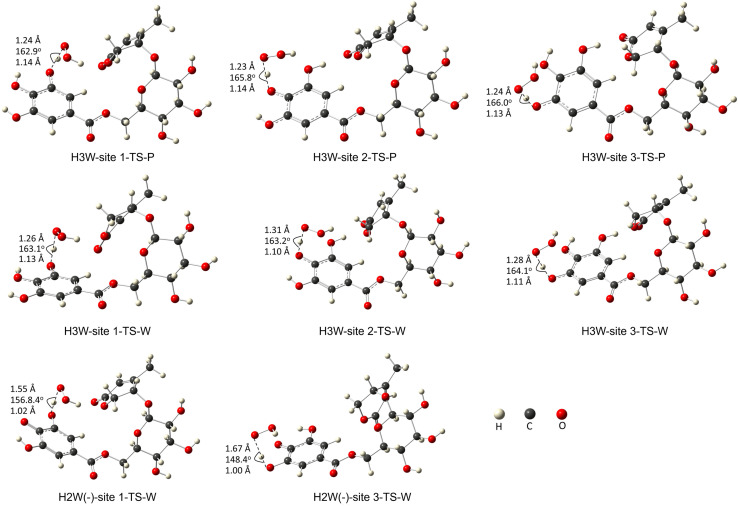
The transition states of the studied species with optimized geometries were performed at the M06-2X/6-311+G(d,p) level of theory. (W: water, P: pentylethanoate).

Fig. 3 depicts the optimized geometry of the transition states of the H_3_W and H_2_W^−^ forms in the studied media, and their cartesian coordinates and energies are also presented in Table S2 (ESI†) and IRC plots for these transition states are also shown in Fig. S1 (ESI†).

**Fig. 4:**
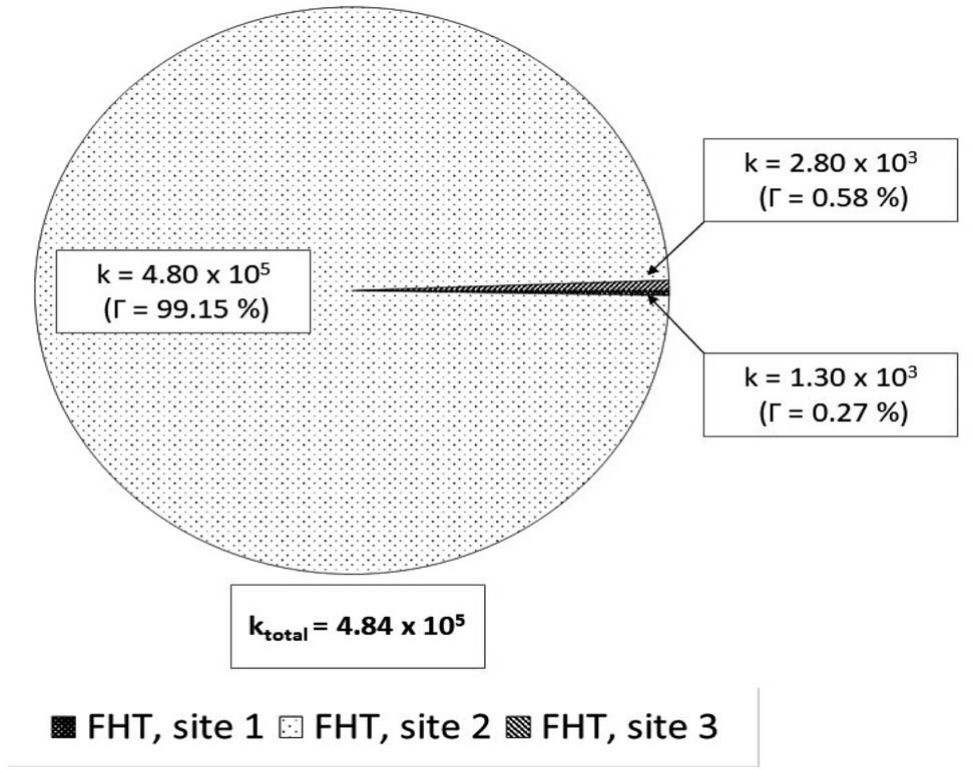
Rate constants (*k*, M^−1^ s^−1^) and branching ratios (*Γ*) of the reactions between Wal (H_3_W) and HOO˙ in lipid solution.

**Fig. 5:**
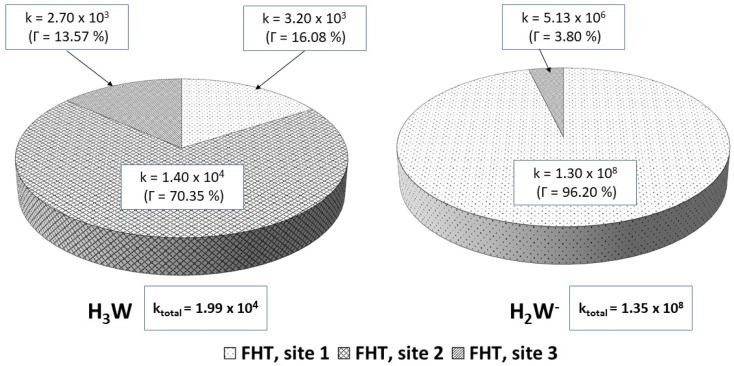
Rate constants (*k*, M^−1^ s^−1^) and branching ratios (*Γ*) of the reactions of the H_3_W and H_2_W^−^ forms with hydroperoxyl radical, in aqueous solution.

For the reactions in each solvent, *k*_total_ (total rate coefficients for each reacting species) and *k*_overall_ (overall rate coefficients) are calculated and displayed in Fig. 4 and 5 and Table 3. Since the neutral species is the only available reactive species, the *k*_total_ and *k*_overall_ values in lipid solution are the same (eqn (5)). The total of the rate constants was determined for each specific route. Conversely, the *k*_total_ values for the non-charged and charged species in aqueous solution were independently determined by adding the rate coefficients *via* the possible paths for each of them. The molar fractions of the various existing states at the physiological pH (7.4) were taken into consideration to compute *k*_overall_ in aqueous solution (eqn (6)).

**Table 3:** Total rate constants (*k*_total_), corrected-by-fraction total rate coefficients (*k*_f_), and overall rate coefficients (*k*_overall_) at physiological pH (7.4)

Species	*f*	*k*_total_ (M^−1^ s^−1^)	*k*_f_ (M^−1^ s^−1^)
H_3_W	0.942	1.99 × 10^4^	1.87 × 10^4^
H_2_W^−^	0.058	1.35 × 10^8^	7.83 × 10^6^
*k*_overall_			7.85 × 10^6^

In lipid medium:

5 *k*_overall_ = *k*_total_ = Σ*k*(FHT-neutral)

In aqueous solution:

6 *k*_overall_ = *f*_(neutral)_·*k*_total_(FHT-H_3_W) + *f*_(anion)_·*k*_total_(FHT-H_2_W^−^)

In lipid solution, compound Wal (*k*_overall_ = 4.84 × 10^5^ M^−1^ s^−1^) reacts approximately 5 and 6 times faster than Trolox (*k*_overall_ = 9.70 × 10^4^ M^−1^ s^−1^, this work) and ascorbic acid (*k*_overall_ = 7.80 × 10^4^ M^−1^ s^−1^, this work). This argument is supported by comparing total rate constants that were calculated using the same methods. Furthermore, the polarity of the solvent enhances the reactivity of the investigated compound. The predicted values for the overall rate coefficients are 7.85 × 10^6^ M^−1^ s^−1^ and 4.84 × 10^5^ M^−1^ s^−1^ corresponding to water and lipid environments, respectively. It reveals that the hydroperoxyl scavenging activity of Wal increases approximately 16 times more rapidly in water solution than in lipid medium. The various existing states of this compound in aqueous solution play a role in another difference in its reactivity. The anion of Wal reacts with the HOO radical 418 times quicker than the neutral species in polar environments. Considering the various reaction sites, the quickest paths for neutral and anion forms are located in the sites 2 and 1, respectively. For a more in depth analysis of the contributions of the reaction channels to Wal's overall hydroperoxyl scavenging behavior, the branching ratios (*Γ*) were computed using eqn (7) and (8).7
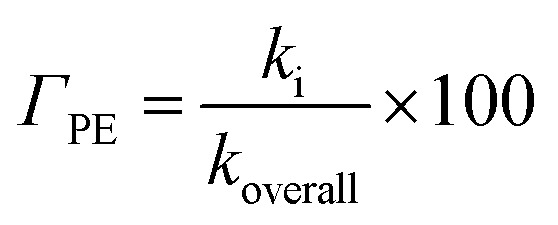
8
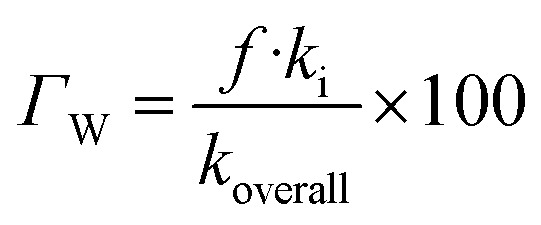


It was discovered that in lipid solution, the FHT reaction from site 2 corresponds to the most important pathway for Wal, accounting for 99.15% of the total contribution, whereas the contributions from sites 1 and 3 are less significant (0.27% and 0.58%, respectively). The anion, which is present in water solution at pH = 7.4, is the only chemical species that contributes to Wal's overall reactivity. On the other hand, neutral species contribute almost nothing. According to the findings presented here, the anion appears to be the crucial species in terms of antiradical activity of Wal. The overall rate coefficient of Wal's reaction with HOO˙ in water medium at pH = 7.4 was compared with those for known antioxidants to get a better idea of how it might function as an antioxidant. Compound Wal (*k*_overall_ = 7.85 × 10^6^ M^−1^ s^−1^) is estimated to be more effective at scavenging HOO˙ radical than Trolox (*k*_overall_ = 1.50 × 10^5^ M^−1^ s^−1^, this work), a commonly used reference antioxidant.

### 3.2. Molecular docking analysis

In this section, in order to illustrate potential molecular modes of action of compound Wal, we conducted molecular docking research on five significant antioxidant enzymes involved in redox homeostasis. First, co-crystallized ligands like S-Warfarin (SWA), protocatecuic acid (PRA), *N*-acetyl-d-glucosamine (NAG), Adenosine-5′-diphosphate (ADP), and Hypoxanthine (HYP) were redocked with CP450, LO, MP, NO, and XO enzymes, respectively, to find the best docking parameters (see Table S3†). The information regarding the validation procedures for molecular docking simulations can be observed in Fig. S2 (ESI†). The optimal position of each docked ligand is determined according to its binding affinity – BA and the root mean square deviation – RMSD (<2.0 Å).^39,40^ Fig. S2† reveals that the overlap between the experimental structures and the docked structures exhibits small RMSD values, which is consistent with what was expected. The same procedures are used in molecular docking simulations to determine how compound Wal under investigation interact with the enzyme binding sites.

**Fig. 6:**
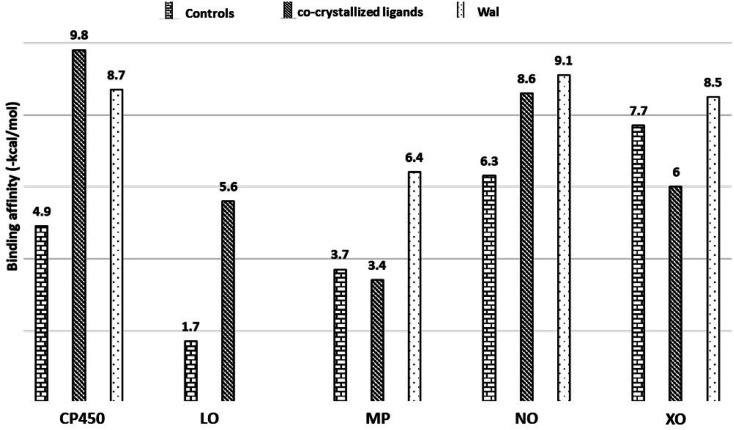
Binding affinities of Wal and control molecules with target enzymes. Controls/co-crystallized ligands for CP450, LO, MP, NO and XO were FLU/SWA, ZIL/PRA, MEL/NAG, DEX/ADP and FEB/HYP, respectively.

The strength of the binding interaction between the ligand and the target enzyme is characterized in terms of BAs (negative value). Fig. 6 shows the BAs of the control ligands and Wal molecule. Since the Wal ligand's positive BA value for the LO receptor is non-significant, it is not displayed.

**Fig. 7:**
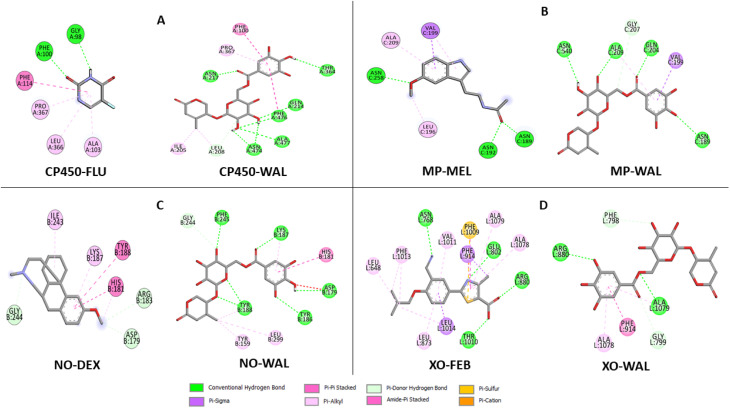
Types of interactions of Wal with target enzymes CP450 (A), MP (B), NO (C) and XO (D) related to generating superoxides.

The data on how the test (Wal) and control (FLU) molecules interacted with the CP450 receptor are shown in Fig. 7A. In the CP450-Wal complex, the active area of interactions is covered by PHE 100, ILE 205, LEU 208, GLN 214, ASN 217, THR 364, PRO 367, ASN 474, PHE 476, and ALA 477 residues. Complex CP450-FLU is stabilized *via* hydrogen bonding and non-covalent interactions with the amino acid residues GLY 98, PHE 100, ALA 103, PHE 114, LEU 366, and PRO 367. The BA of Wal was −8.7 kcal mol^−1^ lower than that of FLU (control), which was −4.9 kcal mol^−1^. Thus, Wal can be regarded as a potent inhibitor of the CP450 enzyme.

In the MP receptor, the control ligand MEL is linked to ANS 189, ANS 192, and ANS 258 amino acids *via* hydrogen bonds (Fig. 7B). It also interacted with ALA 209 and LEU 196 in a pi-alkyl way and with VAL 199 in a pi-sigma way. In a manner analogous, the amino acid residues ASN 189, VAL 199, GLN 204, GLY 207, ALA 209, and ASN 540 make up the active interaction area of the MP-Wal complex. The BA for Wal (−6.4 kcal mol^−1^) is lower than that of MEL (−3.7 kcal mol^−1^). One can draw the conclusion that Wal is also capable of inhibiting the activity of the MP receptor.

In terms of the NO receptor, Fig. 7C illustrates the interaction that takes place between the TYR 159, ASP 179, HIS 181, TYR 186, LYS 187, TYR 188, GLY 244, PHE 245 and LEU 299 residues and Wal ligand with the BA value of −9.1 kcal mol^−1^. Several commonly interacted amino acid residues can be observed in DEX (control), including ASP 179, HIS 181, ARG 183, LYS 187, TYR 188, ILE 243, and GLY 244. The BA value for these interactions is −6.3 kcal mol^−1^. The result that was obtained is in line with the findings of other studies as well.^41,42^ This demonstrates that compound Wal can prevent the action of NO enzyme in producing superoxide.

The FEB and Wal docking results demonstrate significant interactions with the XO enzyme (Fig. 7D). By looking at the interaction sites of Wal and FEB, we found that the results were the same for active sites of XO receptor with the amino acid residues ARG 880, PHE 914, ALA 1078 and ALA 1079. According to our findings, the interactions for FEB are the same as those previously reported.^43,44^ In addition, the control (FEB) has BA of −7.7 kcal mol^−1^, whereas the Wal binding has BA of −8.5 kcal mol^−1^. Therefore, compared to the drug FEB, compound Wal has a higher XO inhibitory action.

Moreover, the binding affinity values at the CP450, MP, NO and XO receptors for Wal are lower than co-crystallized ligands (see Fig. 6), except for CP450-SWA complex, suggesting that compound Wal is more stabilized into the binding pockets of these enzymes *via* the formation of hydrogen bonds and non-covalent interactions.

Compound Wal shows increased binding affinity for target enzymes inhibition mainly through hydrogen bonding interactions. From the list of residues (Fig. 7), seven interact with CP450 (GLN 214, ASN 217, THR 364, ASN 474, PHE 476, and ALA 477), four with MP (ASN 189, GLN 204, ALA 209, and ASN 540), six with NO (ASP 179, TYR 186, LYS 187, TYR 188, and PHE 245) and two with XO (ARG 880 and ALA 1079).

As a result of the preceding discussion, it is possible to draw the conclusion that the compound Wal demonstrates strong binding affinity toward four target receptors (MP, CP450, NO, and XO) and interacts favorably with variety of crucial amino acid residues in these enzymes, indicating that it may have potential antioxidant activity.

## 4. Conclusions

A combination of the density functional theory method at M06-2X/6-311+G(d,p) and molecular docking simulations was efficiently used to evaluate the antiradical and antioxidant activity of the compound Wal. In both aqueous and lipid solutions, the antiradical activity of all species of Wal was investigated to determine whether or not it was caused by the SET or FHT mechanisms. It was proved that FHT is the primary reaction route for both the neutral and anion forms of Wal. The overall rate coefficient values for polar and non-polar environments are predicted to be 7.85 × 10^6^ M^−1^ s^−1^ and 4.84 × 10^5^ M^−1^ s^−1^, respectively. In comparison to lipid medium, the hydroperoxyl scavenging activity of Wal increases approximately 16 times more quickly in aqueous solution. According to the findings concerning kinetic constants, the compound Wal is superior to Trolox in terms of its ability to scavenge HOO˙ from physiological environments (pH = 7.4). In addition, the docking results point to a parallel pathway for the antioxidant properties of Wal, which involves the inhibition of the activity of enzyme families (CP450, MP, NO and XO) that are responsible for producing ROS.

## Author contributions

Phan Tu Quy: conceptualization, investigation, methodology, formal analysis. Nguyen Anh Dzung, Mai Van Bay, Nguyen Van Bon, Doan Manh Dung: investigation, data curation, formal analysis. Pham Cam Nam: formal analysis, writing – review & editing. Nguyen Minh Thong: conceptualization, investigation, methodology, formal analysis, writing – original draft.

## Conflicts of interest

The authors declare that they have no known competing financial interests or personal relationships that could have appeared to influence the work reported in this paper.

## Supplementary Material

RA-012-D2RA05312H-s001
