# Supplementary material for: Insights into antiradical mechanism and pro-oxidant enzyme inhibitor activity of walterolactone A/B 6-O-gallate-β-d-pyranoglucoside originating from Euonymus laxiflorus Champ. using in silico study
Source: RSC Adv. 2022 Oct 19;12(46):29975–82. doi: 10.1039/d2ra05312h (PMC9580510; doi:10.1039/d2ra05312h)
Supplement: RA-012-D2RA05312H-s001 [file RA-012-D2RA05312H-s001.pdf]

Electronic Supplementary Material (ESI) for RSC Advances.
This journal is © The Royal Society of Chemistry 2022

Supplementary Information (SI)

Insights into antiradical mechanism and pro-oxidant enzyme inhibitor activity of walterolactone A/B 6-O-gallate-β-D-pyranoglucoside originated from *Euonymus laxiflorus* Champ. using *in silico* study

Phan Tu Quy[1], Nguyen Anh Dzung[2], Mai Van Bay[3], Nguyen Van Bon[2], Doan Manh Dung[2], Pham Cam Nam[4], and Nguyen Minh Thong[5*]

[1] Department of Natural Sciences & Technology, Tay Nguyen University, Buon Ma Thuot 630000, Vietnam

[2] Institute of Biotechnology and Environment, Tay Nguyen University, Buon Ma Thuot 630000, Vietnam

[3] The University of Danang - University of Science and Education, Danang 550000, Vietnam

[4] The University of Danang - University of Science and Technology, Danang 550000, Vietnam

[5] The University of Danang, Campus in Kon Tum, 704 Phan Dinh Phung, Kon Tum, Vietnam

*Corresponding author: nmthong@kontum.udn.vn. (Nguyen Minh Thong)

Table of Contents

Table S1. The method to calculate rate constant following the conventional transition state theory 3

Table S2. The cartesian coordinates and energies of transition states in the studied media 3

Table S3. Molecular docking parameters for each protein target 13

Figure S1. IRC plots for all transition states related to reaction of $HOO^{\bullet}$ radical with the studied species (W: water, P: pentylethanoate) 14

Figure S2. Parameters obtained in the validation of docking protocols for CP450, LO, MP, NO and XO receptors. The experimental structures (shown in gray) and the docked structures (shown in yellow). 14

Table S1. The method to calculate rate constant following the conventional transition state theory

The rate constant (k) was calculated by using the conventional transition state theory (TST) (at 298.15 K, 1M standard state) according to the equation (1):[1-5]

$$k = \sigma\kappa\frac{k_B T}{h}e^{-(\Delta G^{\neq})/RT} \quad (1)$$

Where: σ is the reaction symmetry number ,[6, 7]
κ contains the tunneling corrections calculated using the Eckart barrier,[8]
k_B is the Boltzmann constant,
h is the Planck constant,
$\Delta G^{\neq}$ is the Gibbs free energy of activation.

The Marcus Theory was used to estimate the reaction barriers of SET reactions.[9-12] The free energy of reaction $\Delta G^{\neq}$ for the SET pathway was computed following the equations (2,3).

$$\Delta G^{\neq}_{SET} = \frac{\lambda}{4}\left(1 + \frac{\Delta G^{0}_{SET}}{\lambda}\right)^2 \quad (2)$$

$$\lambda \approx \Delta E_{SET} - \Delta G^{0}_{SET} \quad (3)$$

where ΔG_{SET} is the Gibbs energy of reaction, ΔE_{SET} is the non-adiabatic energy difference between reactants and vertical products for SET.[13, 14]

For rate constants that were close to the diffusion limit a correction was applied to yield realistic results[15]. The apparent rate constants (k_{app}) were calculated following the Collins–Kimball theory in the solvents at 298.15K;[16] the steady-state Smoluchowski rate constant (k_D) for an irreversible bimolecular diffusion–controlled reaction was calculated following the literature as corroding to equations (4,5).[15, 17]

$$k_{app} = \frac{k_{TST}k_D}{k_{TST} + k_D} \quad (4)$$

$$k_D = 4\pi R_{AB} D_{AB} N_A \quad (5)$$

where R_{AB} is the reaction distance, N_A is the Avogadro constant, and $D_{AB} = D_A + D_B$ (D_{AB} is the mutual diffusion coefficient of the reactants A and B),[16, 18] where D_A or D_B is estimated using the Stokes–Einstein formulation (6).[19, 20]

$$D_{A\ or\ B} = \frac{k_B T}{6\pi\eta a_{A\ or\ B}} \quad (6)$$

η is the viscosity of the solvents (i.e. $\eta(H_2O) = 8.91\times10^{-4}$ Pa s, η(pentyl ethanoate) $= 8.62\times10^{-4}$ Pa s) and a is the radius of the solute.

The kinetic study requires different considerations. Water (dielectric constants, $\varepsilon = 78.35$) and pentyl ethanoate ($\varepsilon = 4.73$) are the *de facto* standard solvents in the literature to mimic the polar and nonpolar environments in the human body.[15, 21-23] Thus, these solvents were used to model the physiological environments. The solvent cage effects were included following the corrections proposed by Okuno,[24] adjusted with the free volume theory according to the Benson correction[15, 25-27] to reduce over-penalizing entropy losses in solution. All transition states were characterized by the existence of only one single imaginary frequency. Intrinsic coordinate calculations (IRCs) were performed to ensure that each transition state is connected correctly with the pre-complex and post-complex. The kinetic calculations were performed by the *Eyringpy* code[28, 29]

Table S2. The cartesian coordinates and energies of transition states in the studied media

**Name**				**H3W-site 1-TS-P**
Cartesian Coordinates				Energy (Hartree)
O	-2.00888000	-0.67005700	-0.74427000	Zero-point correction= 0.429915 (Hartree/Particle)
O	-3.01851800	-3.48720100	1.31247100	Thermal correction to Energy= 0.461660
O	-5.50640300	-2.41293000	0.50409800	Thermal correction to Enthalpy= 0.462604
O	-5.43783100	0.38007300	-0.06691100	Thermal correction to Gibbs Free Energy= 0.366263
O	-3.08772400	1.24597000	-1.36893800	Sum of electronic and zero-point Energies= -1790.323638
O	0.49787700	-1.82319300	0.07437200	Sum of electronic and thermal Energies= -1790.291894
O	1.68618700	-3.49627400	-0.82368400	Sum of electronic and thermal Enthalpies= -1790.290949
O	6.38061800	-1.60345700	-0.89441800	Sum of electronic and thermal Free Energies= -1790.387290
O	3.84531100	1.58803700	1.48103500	
O	6.19722400	0.76587800	0.43320700	
C	-3.15636800	-2.52448100	0.28887500	
C	-4.34113700	-1.61288900	0.56198400	
C	-1.86871900	-1.69724300	0.23081000	
C	-4.37879400	-0.47616600	-0.44116200	
C	-3.03452900	0.24147600	-0.41130300	
C	-0.70138900	-2.56704600	-0.16276200	
C	1.63662800	-2.40820500	-0.30559000	
C	2.82530500	-1.55245500	-0.03636500	
C	2.71325600	-0.37008400	0.67346700	
C	4.06093600	-1.97337300	-0.55352300	
C	5.19521300	-1.19624400	-0.38271500	
C	3.85729600	0.43763600	0.84358500	
C	5.09368100	0.01432600	0.29977900	
H	-3.30696300	-3.01338700	-0.68427400	
H	-4.22736900	-1.18591000	1.56914200	
H	-1.68012200	-1.25410700	1.21925100	
H	-4.53100400	-0.87527600	-1.45187800	
H	-2.85340000	0.65337900	0.59381100	
H	-0.76728600	-2.83385100	-1.22012300	
H	-0.69244500	-3.47717700	0.43938800	
H	-3.87565100	-3.91529800	1.42321300	
H	-6.26624300	-1.84744200	0.68340700	
H	-5.54134500	1.04605100	-0.75562500	
H	1.77161700	-0.03084600	1.08886600	
H	4.13131600	-2.90672800	-1.09938400	
H	7.05746600	-0.94224400	-0.70034800	
H	3.23518900	2.33385900	0.86929700	
H	5.96032500	1.56930800	0.92341500	
C	-2.15890400	2.32911000	-1.27293400	
C	-2.33071400	3.10445800	0.01322600	

C	-0.70675700	1.95559500	-1.49364200	
C	-1.40639800	2.98801300	0.97122900	
H	-0.17415500	2.84718600	-1.83651300	
C	-0.28325400	2.03645500	0.88137100	
H	-1.48817700	3.50758400	1.91886900	
O	-0.03288000	1.46506600	-0.32032600	
O	0.37886100	1.70365700	1.82923300	
H	-0.61828600	1.16363600	-2.23526400	
H	-2.43911200	2.97438600	-2.10899900	
C	-3.56971200	3.92449000	0.15472700	
H	-3.60412100	4.43533700	1.11668100	
H	-3.62903700	4.66393500	-0.64926400	
H	-4.45084200	3.28099700	0.06727700	
O	2.61354600	1.96697400	-1.03113100	
H	1.77374700	1.50210300	-0.83562900	
O	2.68537100	2.92549300	-0.07094400	
**Name**				**H3W- site 2-TS-P**
Cartesian Coordinates				Energy (Hartree)
O	2.24877900	-0.56186400	0.77348300	Zero-point correction= 0.430680 (Hartree/Particle)
O	3.39520900	-3.44536600	-1.11563200	Thermal correction to Energy= 0.462046
O	5.81738600	-2.09667500	-0.57387000	Thermal correction to Enthalpy= 0.462990
O	5.53822200	0.71081400	-0.17657000	Thermal correction to Gibbs Free Energy= 0.367269
O	3.22504800	1.43886400	1.27240600	Sum of electronic and zero-point Energies= -1790.328303
O	-0.16717800	-1.92218500	0.15721800	Sum of electronic and thermal Energies= -1790.296937
O	-1.30533200	-3.71159000	0.88315000	Sum of electronic and thermal Enthalpies= -1790.295993
O	-6.10888300	-2.12832800	0.17082700	Sum of electronic and thermal Free Energies= -1790.391714
O	-3.40102300	1.53188000	-1.13176600	
O	-5.86888100	0.31989300	-0.86771000	
C	3.50668200	-2.39165100	-0.18310400	
C	4.58324000	-1.40708800	-0.60581600	
C	2.15966700	-1.67034100	-0.11540800	
C	4.58034200	-0.20357200	0.31557300	
C	3.18269700	0.40691400	0.34331000	
C	1.07488900	-2.58932700	0.39003300	
C	-1.28687600	-2.59548500	0.43046900	
C	-2.50015600	-1.79000400	0.09955900	
C	-2.34835400	-0.49559800	-0.39350000	
C	-3.75663100	-2.36322800	0.30537900	
C	-4.87973100	-1.61812800	0.00210800	
C	-3.47045800	0.25521800	-0.69803600	
C	-4.76555600	-0.29865200	-0.52271400	
H	3.75601200	-2.78053800	0.81440400	
H	4.36575200	-1.06989200	-1.62993500	
H	1.89285600	-1.32274200	-1.12376300	
H	4.82777900	-0.52153400	1.33614000	
H	2.90321300	0.77542500	-0.65736600	
H	1.19796700	-2.78265600	1.45874300	
H	1.09573300	-3.53383700	-0.15552600	
H	4.28013100	-3.80317500	-1.25408200	

H	6.51069700	-1.48151900	-0.83912500	
H	5.67260000	1.39229100	0.49140100	
H	-1.36348300	-0.07037600	-0.51853200	
H	-3.85133400	-3.36925900	0.69363500	
H	-6.75097900	-1.45637100	-0.10559200	
H	-2.46169300	1.78535500	-1.28547800	
H	-5.98207600	1.32215300	-0.34643600	
C	2.23010900	2.45903200	1.21272300	
C	2.29746900	3.23981100	-0.07996100	
C	0.81300900	2.01323600	1.54006000	
C	1.24485700	3.25494100	-0.90498000	
H	0.29821500	2.83492500	2.04290100	
C	0.06507200	2.40710700	-0.68998100	
H	1.23954700	3.82912400	-1.82385900	
O	0.00957200	1.65058500	0.40299000	
O	-0.83202400	2.32445000	-1.50992000	
H	0.82287100	1.13773600	2.18441800	
H	2.52635900	3.13446500	2.01944000	
C	3.57599600	3.95130100	-0.37460000	
H	3.49193300	4.56530300	-1.27085700	
H	3.86999600	4.57863300	0.47140100	
H	4.37477700	3.21882600	-0.52922000	
O	-4.84258600	2.12962700	1.14437100	
H	-4.15246400	2.28468600	0.46929800	
O	-6.00855900	2.25315500	0.46243600	
**Name**				**H3W- site 3-TS-P**
Cartesian Coordinates				Energy (Hartree)
O	2.21615500	-0.64140900	0.75556000	Zero-point correction= 0.429928 (Hartree/Particle)
O	3.38993500	-3.47956400	-1.18165500	Thermal correction to Energy= 0.461663
O	5.81210100	-2.24484200	-0.41125400	Thermal correction to Enthalpy= 0.462607
O	5.60433200	0.54851000	0.09198500	Thermal correction to Gibbs Free Energy= 0.365363
O	3.21723400	1.29386600	1.42553800	Sum of electronic and zero-point Energies= -1790.323010
O	-0.19440600	-1.84359000	-0.09824700	Sum of electronic and thermal Energies= -1790.291275
O	-1.48294700	-3.46124700	0.76728600	Sum of electronic and thermal Enthalpies= -1790.290331
O	-6.10701300	-1.37791400	0.04138200	Sum of electronic and thermal Free Energies= -1790.387575
O	-3.11035600	1.78613200	-1.70994400	
O	-5.59876600	1.05413300	-1.10380400	
C	3.46987000	-2.47173800	-0.19650700	
C	4.60728100	-1.50984800	-0.49639500	
C	2.14959400	-1.70079900	-0.19193700	
C	4.58236200	-0.34774000	0.47706700	
C	3.20826900	0.31344800	0.44101800	
C	0.99005700	-2.58715100	0.19240200	
C	-1.36842300	-2.38705000	0.23442300	
C	-2.49817000	-1.48729700	-0.13573000	
C	-2.22894500	-0.26888400	-0.78681500	
C	-3.78785600	-1.85780700	0.17767300	
C	-4.86658300	-1.01599200	-0.17481300	
C	-3.26269000	0.58485200	-1.10722300	

C	-4.58204800	0.22068800	-0.81082800	
H	3.63211200	-2.91216400	0.79747200	
H	4.47447200	-1.11941800	-1.51611300	
H	1.97328700	-1.29395000	-1.19825500	
H	4.74765000	-0.71918700	1.49616400	
H	3.01140200	0.74331100	-0.55495700	
H	1.02694600	-2.82985800	1.25703000	
H	1.00251000	-3.50813600	-0.39300200	
H	4.26825900	-3.86838900	-1.26884100	
H	6.54276300	-1.64403000	-0.59720800	
H	5.72118700	1.19224000	0.79934800	
H	-1.21059500	0.01204500	-1.01367300	
H	-3.99949200	-2.79555400	0.67635000	
H	-6.62569100	-0.64739200	0.73318000	
H	-2.16045900	2.02105400	-1.75941300	
H	-5.24192200	1.81020600	-1.59561000	
C	2.24927200	2.33970700	1.35689200	
C	2.47413700	3.23399000	0.15839100	
C	0.79720000	1.90482900	1.47350000	
C	1.53294400	3.32779800	-0.78730100	
H	0.23568000	2.70590900	1.95972500	
C	0.33635300	2.47411700	-0.79431800	
H	1.64007600	3.97941000	-1.64642800	
O	0.14550700	1.63366500	0.22028400	
O	-0.44763200	2.46401200	-1.72549800	
H	0.70932500	0.99057100	2.05481700	
H	2.46114500	2.92803400	2.25333700	
C	3.77390300	3.96488800	0.09306300	
H	3.80321900	4.64928300	-0.75440400	
H	3.94690600	4.52220900	1.01792900	
H	4.59353700	3.24698000	-0.01098400	
O	-6.91934500	0.16813700	1.61690900	
O	-5.79748400	0.92726200	1.75283100	
H	-5.83000400	1.53955300	0.99718300	
**Name**				**H2W(-)-site 1-TS-W**
Cartesian Coordinates				Energy (Hartree)
O	-1.96762800	-0.70465000	-0.68498500	Zero-point correction= 0.416790 (Hartree/Particle)
O	-3.40132700	-3.31087900	1.40042700	Thermal correction to Energy= 0.448808
O	-5.71169100	-1.97995600	0.40701900	Thermal correction to Enthalpy= 0.449753
O	-5.26221400	0.78976200	-0.19580800	Thermal correction to Gibbs Free Energy= 0.352519
O	-2.77292900	1.27167000	-1.48419900	Sum of electronic and zero-point Energies= -1789.907968
O	0.33129500	-2.00738500	0.24143400	Sum of electronic and thermal Energies= -1789.875950
O	1.41858700	-3.61115200	-0.88490600	Sum of electronic and thermal Enthalpies= -1789.875006
O	6.03606300	-1.43986400	-1.38711300	Sum of electronic and thermal Free Energies= -1789.972240
O	3.75536000	1.12314300	1.93782100	
O	5.98258300	0.60561100	0.35356900	
C	-3.37721800	-2.37342300	0.33843500	
C	-4.46027300	-1.32174900	0.52225700	
C	-2.00455000	-1.70187600	0.33303000	

C	-4.31309300	-0.21308700	-0.50654900	
C	-2.89415000	0.34124100	-0.45220900	
C	-0.90843900	-2.69105200	0.02296900	
C	1.43918300	-2.56215700	-0.26715700	
C	2.64118000	-1.75132700	-0.02331300	
C	2.62895900	-0.70759700	0.89076200	
C	3.80210900	-2.02237600	-0.78887100	
C	4.90449200	-1.22838500	-0.64647400	
C	3.75660700	0.09697500	1.04907800	
C	4.94537100	-0.12086400	0.27505600	
H	-3.53176300	-2.87988000	-0.62337900	
H	-4.35710800	-0.88717500	1.52542300	
H	-1.82422200	-1.23742300	1.31222200	
H	-4.48509700	-0.61647100	-1.51169800	
H	-2.69862400	0.79505900	0.52953400	
H	-0.97770500	-3.01172200	-1.01804600	
H	-0.96561300	-3.55849200	0.68145600	
H	-4.28722700	-3.69229500	1.43611100	
H	-6.40520700	-1.36430900	0.67233700	
H	-5.27098900	1.43314700	-0.91527900	
H	1.74804500	-0.48557400	1.47947800	
H	3.80819200	-2.83788700	-1.50285500	
H	6.66072500	-0.74231600	-1.12781400	
H	3.63336200	1.98564600	1.41511200	
C	-1.77540000	2.29446300	-1.37565000	
C	-1.99683500	3.16281800	-0.16015000	
C	-0.33960000	1.81890800	-1.46196600	
C	-1.12515800	3.11074400	0.85282200	
H	0.27131700	2.64296100	-1.83653100	
C	-0.02679800	2.13680200	0.90062600	
H	-1.24231100	3.71472100	1.74477500	
O	0.23247600	1.40449100	-0.20058600	
O	0.62283100	1.91829100	1.90071100	
H	-0.24471300	0.96054600	-2.12325100	
H	-1.94575900	2.90273400	-2.26665400	
C	-3.21845100	4.01681900	-0.15237600	
H	-3.29782900	4.59101300	0.76992800	
H	-3.20255800	4.69768900	-1.00821000	
H	-4.10675200	3.38617100	-0.25960400	
O	3.05935800	1.92710200	-0.72565300	
H	2.16192600	1.56451000	-0.57142000	
O	3.23877200	2.88007600	0.20932400	
**Name**				**H2W(-)-site 3-TS-W**
Cartesian Coordinates				Energy (Hartree)
O	1.96983000	-0.72854600	0.72529100	Zero-point correction= 0.417370 (Hartree/Particle)
O	3.26834100	-3.51876800	-1.18666200	Thermal correction to Energy= 0.449845
O	5.58878800	-2.50947700	0.08160700	Thermal correction to Enthalpy= 0.450790
O	5.47054800	0.28662500	0.65770700	Thermal correction to Gibbs Free Energy= 0.350642
O	2.89677800	1.16798800	1.55679200	Sum of electronic and zero-point Energies= -1789.871796

O	-0.38708400	-1.71029100	-0.32279300	Sum of electronic and thermal Energies= -1789.839320
O	-1.74810800	-3.40801500	0.20299900	Sum of electronic and thermal Enthalpies= -1789.838376
O	-3.24941700	2.24894900	-1.15876700	Sum of electronic and thermal Free Energies= -1789.938523
O	-5.75635600	1.38962100	-0.84265400	
C	3.23869800	-2.57880200	-0.13271100	
C	4.46834700	-1.68361300	-0.16916100	
C	1.97446000	-1.73059500	-0.27870200	
C	4.35326100	-0.55603700	0.84436800	
C	3.04346000	0.17884400	0.59341100	
C	0.73103500	-2.56529700	-0.08995600	
C	-1.60240400	-2.24618200	-0.11507500	
C	-2.69080900	-1.27142100	-0.30480700	
C	-2.41446700	0.07134900	-0.66206100	
C	-3.99897700	-1.69571200	-0.09020900	
C	-5.05680400	-0.80775000	-0.25286000	
C	-3.45202900	0.95039800	-0.81317100	
C	-4.83128400	0.55603000	-0.65334200	
H	3.21048400	-3.09021900	0.83967400	
H	4.54651800	-1.24041000	-1.17245700	
H	1.95862900	-1.26310100	-1.27389900	
H	4.33172500	-0.96808500	1.86066300	
H	3.05126400	0.60520700	-0.42115400	
H	0.69007400	-2.95533000	0.93020500	
H	0.72345500	-3.39898700	-0.79371500	
H	4.11909600	-3.97105300	-1.14883200	
H	6.38333500	-1.96859800	0.01400300	
H	5.50275500	0.91672700	1.38492700	
H	-1.39489800	0.40130300	-0.81725800	
H	-4.20095900	-2.71705600	0.20970300	
H	-4.13785200	2.64008900	-1.20737200	
C	2.01416900	2.25203100	1.24011000	
C	2.69498300	3.22147300	0.29892100	
C	0.65028300	1.85586100	0.70233200	
C	2.31526900	3.26327200	-0.98229600	
H	-0.03078600	2.69813100	0.84506500	
C	1.32010400	2.33072700	-1.54648400	
H	2.77952000	3.92906600	-1.69995600	
O	0.66245400	1.52405700	-0.69562400	
O	1.12426000	2.21528300	-2.73287800	
H	0.26098100	0.97446100	1.20545500	
H	1.85040600	2.74463200	2.20070100	
C	3.78691100	4.06524700	0.86950900	
H	4.28849900	4.65046600	0.10025400	
H	3.37481200	4.74175200	1.62384900	
H	4.51991000	3.43191400	1.37583000	
O	-6.32955500	-1.19783800	-0.04550500	
H	-6.76410600	-0.49679600	0.52052800	
O	-6.74914000	0.40718800	1.92009200	
O	-5.40892100	0.46902100	2.11989300	
H	-5.14745200	-0.40944300	2.44090900	

**Name**				**H3W- site 1-TS-W**	
Cartesian Coordinates				Energy (Hartree)	
O	-2.00068900	-0.69722500	-0.73492400	Zero-point correction=	0.429281 (Hartree/Particle)
O	-3.12339100	-3.49326500	1.29364500	Thermal correction to Energy=	0.461127
O	-5.57702600	-2.32910100	0.44267500	Thermal correction to Enthalpy=	0.462071
O	-5.38131800	0.49828900	-0.04552300	Thermal correction to Gibbs Free Energy=	0.365857
O	-3.00433200	1.24211900	-1.39945400	Sum of electronic and zero-point Energies=	-1790.345686
O	0.44487600	-1.86313500	0.05991400	Sum of electronic and thermal Energies=	-1790.313839
O	1.63951300	-3.58804000	-0.72005500	Sum of electronic and thermal Enthalpies=	-1790.312895
O	6.31657200	-1.63858300	-0.93985400	Sum of electronic and thermal Free Energies=	-1790.409109
O	3.78090000	1.51846400	1.53591300		
O	6.13947300	0.73738400	0.40755200		
C	-3.22330600	-2.51644300	0.27265400		
C	-4.38293000	-1.56998600	0.54357100		
C	-1.91154900	-1.73086100	0.24179900		
C	-4.37007200	-0.41054100	-0.43802200		
C	-2.99973900	0.25582600	-0.41349700		
C	-0.75766400	-2.62237000	-0.13968500		
C	1.58774000	-2.46725200	-0.26098100		
C	2.77463100	-1.61086500	-0.01248800		
C	2.66874900	-0.43638400	0.70728600		
C	4.00665400	-2.01834100	-0.55185400		
C	5.13433900	-1.23350600	-0.39607300		
C	3.80881800	0.37570700	0.87096200		
C	5.04147600	-0.02483900	0.29830500		
H	-3.37522900	-2.99571000	-0.70341600		
H	-4.27686600	-1.17144200	1.56113400		
H	-1.72953300	-1.28990800	1.23168200		
H	-4.55145000	-0.78366400	-1.45321800		
H	-2.79867200	0.67715400	0.58139600		
H	-0.83242900	-2.91669300	-1.18866700		
H	-0.73207400	-3.50992900	0.49271600		
H	-3.97146700	-3.95087300	1.34812200		
H	-6.30724300	-1.79881000	0.78264000		
H	-5.45313300	1.18253200	-0.72265800		
H	1.73309800	-0.10336900	1.13791000		
H	4.08160300	-2.94487900	-1.10863500		
H	7.00997300	-0.99430900	-0.73998800		
H	3.31165900	2.30128700	0.86957100		
H	5.92041800	1.54132300	0.90669600		
C	-2.08813500	2.33704500	-1.28272800		
C	-2.29566100	3.11162500	-0.00262500		
C	-0.62947000	1.98477500	-1.47816700		
C	-1.36137900	3.06255900	0.95360400		
H	-0.10040600	2.88066700	-1.81024000		
C	-0.20118900	2.16454300	0.87818200		
H	-1.46492500	3.60202500	1.88758400		
O	0.03143300	1.51656200	-0.28111300		
O	0.51786900	1.92708400	1.82472300		

H	-0.50613900	1.19037900	-2.21100000	
H	-2.36028100	2.98194900	-2.12112600	
C	-3.57165500	3.87047200	0.12759100	
H	-3.64150800	4.37342900	1.09133100	
H	-3.64987000	4.60683600	-0.67743700	
H	-4.41710500	3.18362200	0.01794100	
O	2.72633100	1.91851700	-1.04297400	
H	1.84341700	1.52819900	-0.86837300	
O	2.85106800	2.91133000	-0.12689600	
**Name**				**H3W- site 2-TS-W**
Cartesian Coordinates				Energy (Hartree)
O	2.00344900	-0.65509000	0.69047300	Zero-point correction= 0.429627 (Hartree/Particle)
O	3.85138200	-3.16246700	-1.17763600	Thermal correction to Energy= 0.461336
O	5.92411400	-1.48333800	-0.19046600	Thermal correction to Enthalpy= 0.462280
O	5.09808600	1.22953300	0.24091800	Thermal correction to Gibbs Free Energy= 0.366573
O	2.52253200	1.42859300	1.44896500	Sum of electronic and zero-point Energies= -1790.347737
O	-0.05947400	-2.19844800	-0.26227100	Sum of electronic and thermal Energies= -1790.316027
O	-1.14286100	-3.86639000	0.77265000	Sum of electronic and thermal Enthalpies= -1790.315083
O	-5.72459300	-1.59927300	1.26012300	Sum of electronic and thermal Free Energies= -1790.410790
O	-3.35920500	1.00870700	-1.91011000	
O	-5.58257000	0.52183300	-0.43846000	
C	3.66007300	-2.17586900	-0.17924300	
C	4.60471000	-1.00247500	-0.38829000	
C	2.21943700	-1.68175600	-0.27400800	
C	4.27251900	0.12864400	0.57102100	
C	2.79672200	0.49472500	0.44905700	
C	1.22189600	-2.77615300	0.01800800	
C	-1.15038200	-2.80528500	0.18955400	
C	-2.36582600	-1.98209300	-0.07251200	
C	-2.28855500	-0.92973400	-0.97918300	
C	-3.52111600	-2.24772600	0.66965500	
C	-4.60366900	-1.40160900	0.53287600	
C	-3.37244600	-0.07845000	-1.11864700	
C	-4.54889400	-0.29677700	-0.35683200	
H	3.83071500	-2.60063400	0.81845300	
H	4.48804900	-0.63979200	-1.41816100	
H	2.04089800	-1.28377600	-1.28247400	
H	4.45502400	-0.20072500	1.60097800	
H	2.57663600	0.89245700	-0.55192600	
H	1.27033800	-3.07359500	1.06736400	
H	1.38180200	-3.64168300	-0.62531300	
H	4.78025000	-3.42450500	-1.15776200	
H	6.54344900	-0.79524700	-0.46077500	
H	5.02642500	1.88376200	0.94645500	
H	-1.38678700	-0.73919400	-1.54334200	
H	-3.55741100	-3.07146300	1.37134900	
H	-6.35744300	-0.89343000	1.05422200	
H	-2.43179500	1.22579600	-2.14823300	
H	-5.27565900	1.49982200	-0.03912300	

C	1.42166800	2.32819800	1.27661000	
C	1.63622800	3.25483700	0.10188000	
C	0.04721500	1.69019300	1.23270200	
C	0.84121900	3.15786500	-0.97038800	
H	-0.68120500	2.42241400	1.58636400	
C	-0.15905800	2.09453700	-1.10927900	
H	0.94850200	3.81014600	-1.82871800	
O	-0.37432400	1.27359300	-0.08230100	
O	-0.76556900	1.89642400	-2.15174400	
H	0.00122700	0.79797800	1.85286900	
H	1.44824100	2.92847000	2.18864100	
C	2.75046100	4.23842600	0.21203700	
H	2.84791800	4.83446100	-0.69444600	
H	2.57515900	4.89826500	1.06689100	
H	3.69105700	3.71294900	0.40104000	
O	-3.51108700	1.83384600	1.02056800	
H	-2.84327900	2.01629400	0.33164500	
O	-4.64804400	2.44550600	0.60852600	
**Name**				**H3W- site 3-TS-W**
Cartesian Coordinates				Energy (Hartree)
O	2.15462600	-0.67130800	0.77361600	Zero-point correction= 0.429329 (Hartree/Particle)
O	3.32971700	-3.51079100	-1.16394400	Thermal correction to Energy= 0.461267
O	5.76371600	-2.28777400	-0.33525800	Thermal correction to Enthalpy= 0.462211
O	5.53487100	0.53817700	0.10186200	Thermal correction to Gibbs Free Energy= 0.364674
O	3.14644300	1.26264300	1.46151900	Sum of electronic and zero-point Energies= -1790.343708
O	-0.24773900	-1.81472200	-0.07082700	Sum of electronic and thermal Energies= -1790.311770
O	-1.57196100	-3.50344300	0.57054600	Sum of electronic and thermal Enthalpies= -1790.310826
O	-6.15698300	-1.24626700	0.02168700	Sum of electronic and thermal Free Energies= -1790.408363
O	-3.07637800	1.86572000	-1.71616200	
O	-5.61678900	1.14153200	-1.09650300	
C	3.41026100	-2.50306900	-0.17174000	
C	4.55912100	-1.54929500	-0.46032200	
C	2.09564800	-1.72542600	-0.18340700	
C	4.52515700	-0.37086500	0.49738100	
C	3.15047500	0.28916200	0.46205300	
C	0.92369800	-2.60179100	0.18193300	
C	-1.43380000	-2.37962400	0.13891800	
C	-2.54779300	-1.46037100	-0.21192000	
C	-2.26034800	-0.23368700	-0.84237800	
C	-3.84241500	-1.81295200	0.10252300	
C	-4.89495800	-0.93436800	-0.22161700	
C	-3.27147000	0.65320700	-1.13582600	
C	-4.59978700	0.31195700	-0.83574900	
H	3.55601400	-2.95065000	0.81998000	
H	4.45296800	-1.17602500	-1.48749800	
H	1.93679900	-1.30559000	-1.18636500	
H	4.70070600	-0.72714400	1.51962400	
H	2.94778000	0.72297100	-0.52776200	
H	0.95915000	-2.87984100	1.23755000	

H	0.90028000	-3.49735900	-0.43925200	
H	4.18463500	-3.95720200	-1.20117500	
H	6.48974000	-1.74754400	-0.66877100	
H	5.63585300	1.19889400	0.79798200	
H	-1.24258100	0.02859600	-1.09502700	
H	-4.07512700	-2.75164800	0.58997100	
H	-6.54000900	-0.58219200	0.82471700	
H	-2.11550700	2.05296800	-1.77125800	
H	-5.27620900	1.96360400	-1.48320800	
C	2.23187300	2.35832000	1.35435400	
C	2.51276000	3.20762600	0.13644100	
C	0.76333700	1.99561500	1.45404800	
C	1.60446400	3.27828700	-0.84368200	
H	0.22429000	2.85409400	1.85847300	
C	0.39778200	2.44577200	-0.85534400	
H	1.75247800	3.89278400	-1.72357900	
O	0.14380700	1.66365500	0.19447000	
O	-0.35397400	2.39774000	-1.81708000	
H	0.61090100	1.13142300	2.09563400	
H	2.45754900	2.95634700	2.24012100	
C	3.81879900	3.92439700	0.09586200	
H	3.91920600	4.51663800	-0.81292100	
H	3.91444000	4.57438500	0.97022900	
H	4.63742100	3.19978300	0.14229400	
O	-6.63662000	0.21548200	1.82253100	
O	-5.35959300	0.65270300	1.98220900	
H	-5.31274000	1.49853600	1.50175600	

Table S3. Molecular docking parameters for each protein target

Protein/PDB ID	Ligand	Coordinates of the Grid Box Center	Grid Box Size
Cytochrome P450/ 1OG5	S-Warfarin	center_x = -20.257<br>center_y = 86.991<br>center_z = 38.581	size_x = 20<br>size_y = 18<br>size_z = 22
Lipoxygenase/ 1N8Q	Protocatecuic acid	center_x = 21.864<br>center_y = 2.184<br>center_z = 18.909	size_x = 22<br>size_y = 16<br>size_z = 10
Myeloperoxidase/ 1DNU	N-Acetyl-D-glucosamine	center_x = 39.817<br>center_y = -38.635<br>center_z = -5.308	size_x = 9<br>size_y = 12<br>size_z = 7
NADPH oxidase/ 2CDU	Adenosine-5’-diphosphate	center_x = 1.687<br>center_y = 9.885<br>center_z = 54.962	size_x = 15<br>size_y = 7<br>size_z = 16

Xanthine oxidase/ 3NRZ	Hypoxanthine	center_x = 89.018<br>center_y = 9.4501<br>center_z = 18.290	size_x = 7<br>size_y = 10<br>size_z = 9

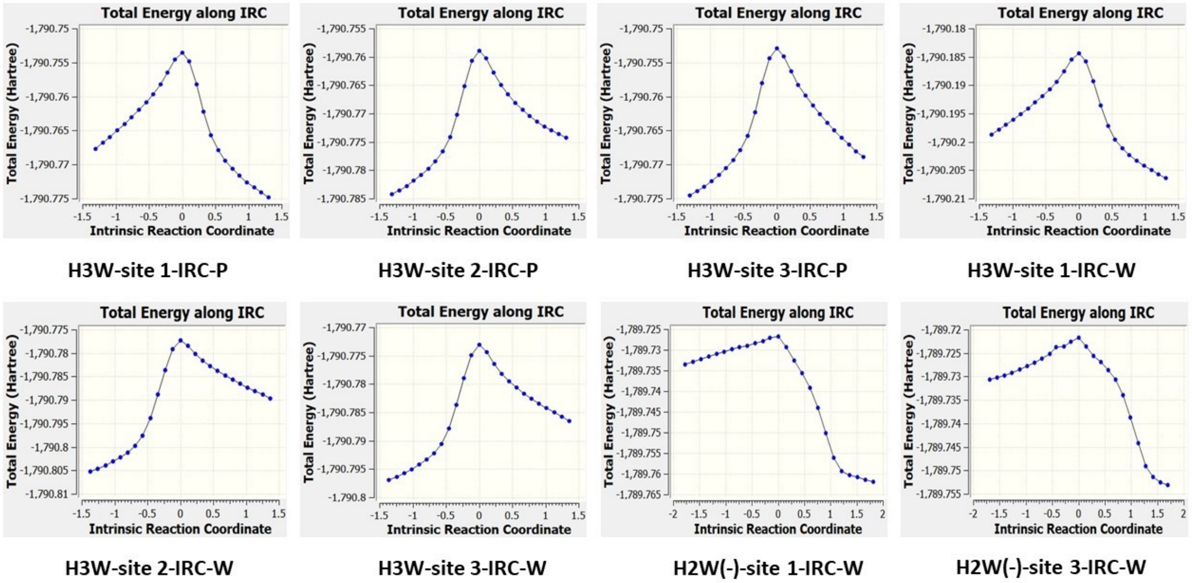

Figure S1. IRC plots for all transition states related to reaction of $HOO^{\bullet}$ radical with the studied species. (W: water, P: pentylethanoate)

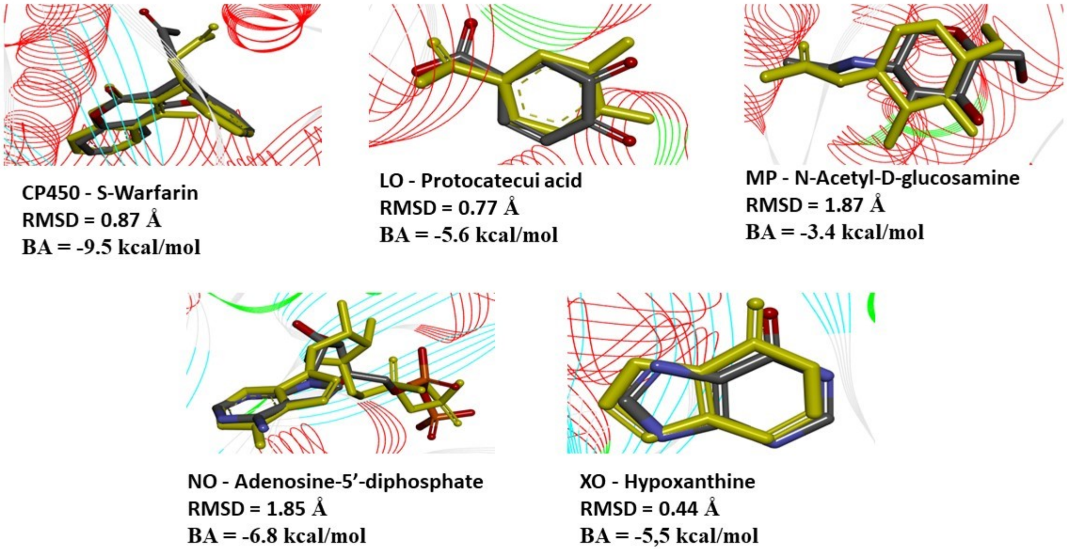

Figure S2. Parameters obtained in the validation of docking protocols for CP450, LO, MP, NO and XO receptors. The experimental structures (shown in gray) and the docked structures (shown in yellow).

References

1. M. G. Evans and M. Polanyi, *Trans. Faraday Soc.*, 1935, **31**, 875-894.
2. H. Eyring, *J. Chem. Phys.*, 1935, **3**, 107-115.
3. D. G. Truhlar, W. L. Hase and J. T. Hynes, *J. Phys. Chem.*, 1983, **87**, 2664-2682.
4. T. Furuncuoglu, I. Ugur, I. Degirmenci and V. Aviyente, *Macromolecules*, 2010, **43**, 1823-1835.
5. E. Vélez, J. Quijano, R. Notario, E. Pabón, J. Murillo, J. Leal, E. Zapata and G. Alarcón, *J. Phys. Org. Chem.*, 2009, **22**, 971-977.
6. E. Pollak and P. Pechukas, *J. Am. Chem. Soc.*, 1978, **100**, 2984-2991.
7. A. Fernández-Ramos, B. A. Ellingson, R. Meana-Pañeda, J. M. Marques and D. G. Truhlar, *Theor. Chem. Acc.*, 2007, **118**, 813-826.
8. C. Eckart, *Phy. Rev.*, 1930, **35**, 1303.
9. R. A. Marcus, *Annu. Rev. Phys. Chem.*, 1964, **15**, 155-196.
10. R. A. Marcus, *Rev. Mod. Phys.*, 1993, **65**, 599.
11. Y. Lu, A. Wang, P. Shi and H. Zhang, *PloS one*, 2017, **12**, e0169773.
12. Y. Lu, A. Wang, P. Shi, H. Zhang and Z. Li, *PloS one*, 2015, **10**, e0133259.
13. S. F. Nelsen, S. C. Blackstock and Y. Kim, *J. Am. Chem. Soc.*, 1987, **109**, 677-682.
14. S. F. Nelsen, M. N. Weaver, Y. Luo, J. R. Pladziewicz, L. K. Ausman, T. L. Jentzsch and J. J. O'Konek, *J. Phys. Chem. A*, 2006, **110**, 11665-11676.
15. A. Galano and J. R. Alvarez-Idaboy, *J. Comput. Chem.*, 2013, **34**, 2430-2445.
16. F. C. Collins and G. E. Kimball, *J. Colloid Sci.* , 1949, **4**, 425-437.
17. M. Von Smoluchowski, *Z. Phys. Chem*, 1917, **92**, 129-168.
18. D. G. Truhlar, *J. Chem. Educ.*, 1985, **62**, 104.
19. A. Einstein, *Ann. Phys.*, 1905, **17**, 549-560.
20. G. G. Stokes, *Mathematical and Physical Papers*, University Press, Cambridge, 1905.
21. A. Galano and J. Raúl Alvarez-Idaboy, *Int. J. Quantum Chem.*, 2019, **119**, e25665.
22. Q. V. Vo, T. V. Gon, M. V. Bay and A. Mechler, *J. Phys. Chem. B*, 2019, **123**, 10672-10679.
23. Q. V. Vo and A. Mechler, *J. Chem. Inf. Model.*, 2020, **60**, 316-321.
24. Y. Okuno, *Chem.: Eur. J.*, 1997, **3**, 212-218.
25. S. Benson, *The foundations of chemical kinetics:* , Malabar, Florida, 1982.
26. C. Iuga, J. R. Alvarez-Idaboy and A. Vivier-Bunge, *J. Phys. Chem. B*, 2011, **115**, 12234-12246.
27. J. R. Alvarez-Idaboy, L. Reyes and N. Mora-Diez, *Org. Biomol. Chem.*, 2007, **5**, 3682-3689.
28. E. Dzib, J. L. Cabellos, F. Ortíz-Chi, S. Pan, A. Galano and G. Merino, *Int. J. Quantum Chem.*, 2019, **119**, e25686.
29. E. Dzib, J. L. Cabellos, F. Ortiz-Chi, S. Pan, A. Galano and G. Merino, *Eyringpy 1.0.2*, 2018, Cinvestav, Mérida, Yucatán.
